# LGR5 receptor promotes cell–cell adhesion in stem cells and colon cancer cells via the IQGAP1–Rac1 pathway

**DOI:** 10.1074/jbc.M117.786798

**Published:** 2017-07-24

**Authors:** Kendra S. Carmon, Xing Gong, Jing Yi, Ling Wu, Anthony Thomas, Catherine M. Moore, Ikuo Masuho, David J. Timson, Kirill A. Martemyanov, Qingyun J. Liu

**Affiliations:** From the ‡ Brown Foundation Institute of Molecular Medicine and Texas Therapeutics Institute, University of Texas Health Science Center, Houston, Texas 77030,; § Department of Cancer Biology, University of Texas M. D. Anderson Cancer Center, Houston, Texas 77030,; ¶ School of Biological Sciences, Queen's University Belfast, Medical Biology Centre, 97 Lisburn Road, Belfast BT9 7BL, Ireland, United Kingdom,; ‖ Department of Neuroscience, The Scripps Research Institute, Jupiter, Florida 33458, and; ** School of Pharmacy and Biomolecular Sciences, University of Brighton, Huxley Building, Lewes Road, Brighton BN2 4GJ, United Kingdom

**Keywords:** 7-helix receptor, adhesion, cancer stem cells, G protein-coupled receptor (GPCR), intestine

## Abstract

Leucine-rich repeat-containing G protein–coupled receptor 5 (LGR5) is a *bona fide* marker of adult stem cells in several epithelial tissues, most notably in the intestinal crypts, and is highly up-regulated in many colorectal, hepatocellular, and ovarian cancers. LGR5 activation by R-spondin (RSPO) ligands potentiates Wnt/β-catenin signaling *in vitro*; however, deletion of LGR5 in stem cells has little or no effect on Wnt/β-catenin signaling or cell proliferation *in vivo*. Remarkably, modulation of LGR5 expression has a major impact on the actin cytoskeletal structure and cell adhesion in the absence of RSPO stimulation, but the molecular mechanism is unclear. Here, we show that LGR5 interacts with IQ motif-containing GTPase-activating protein 1 (IQGAP1), an effector of Rac1/CDC42 GTPases, in the regulation of actin cytoskeleton dynamics and cell–cell adhesion. Specifically, LGR5 decreased levels of IQGAP1 phosphorylation at Ser-1441/1443, leading to increased binding of Rac1 to IQGAP1 and thus higher levels of cortical F-actin and enhanced cell–cell adhesion. LGR5 ablation in colon cancer cells and crypt stem cells resulted in loss of cortical F-actin, reduced cell–cell adhesion, and disrupted localization of adhesion-associated proteins. No evidence of LGR5 coupling to any of the four major subtypes of heterotrimeric G proteins was found. These findings suggest that LGR5 primarily functions via the IQGAP1–Rac1 pathway to strengthen cell–cell adhesion in normal adult crypt stem cells and colon cancer cells.

## Introduction

Leucine-rich repeat-containing G protein–coupled receptor 5 (LGR5)^3^ has emerged as an authentic marker of adult stem cells in several epithelial tissues, including the intestine, liver, skin, stomach, and ovarian epithelium (1, 2). LGR5 and its closely related homologs LGR4 and LGR6 consist of a large extracellular domain (ECD) with 17 leucine-rich repeats and a seven-transmembrane (7TM) domain typical of the rhodopsin family of G protein–coupled receptors (GPCRs) (3, 4). Several studies have shown that the four R-spondin (RSPO1–4) growth factors can bind to LGR4–6 and potentiate canonical/β-catenin-dependent Wnt signaling in some cell types (5–7). Although LGR4–6 are homologous to GPCRs and thus classified into this family, their function in modulation of Wnt signaling is independent of heterotrimeric G proteins (5, 6, 8). For LGR4, it was shown that RSPO–LGR4 potentiates Wnt signaling by inhibiting the two E3 ubiquitin ligases RNF43 and ZNRF3, which otherwise antagonize Wnt signaling through ubiquitination and subsequent degradation of Wnt receptors (9, 10). More recently, we showed that RSPO–LGR4 also functions through the IQGAP1 scaffolding protein to potentiate Wnt signaling via supercomplex formation with the Wnt receptor complex (11). The interaction of LGR4 with IQGAP1 enhances levels of β-catenin through MEK1/2-mediated phosphorylation of LRP6 and promotes association with cytoskeletal components to regulate focal adhesion assembly and cell migration (11).

In contrast, the exact roles and mechanism of LGR5 in the potentiation of Wnt signaling and the regulation of cellular functions remain enigmatic. LGR5 is currently the most recognized marker of adult stem cells in multiple epithelial tissues. Knock-out (KO) of LGR5 in the mouse had no effect on Wnt/β-catenin signaling or the self-renewal of stem cells in the intestine (6, 12, 13) and liver (14), whereas LGR4 is absolutely essential. Conversely, overexpression or knock-out of LGR5 in several colon and liver cancer cell lines led to significant changes in actin cytoskeleton structures and cell–cell adhesion in the absence of RSPO stimulation (15, 16). Furthermore, overexpression of endocytosis-impaired LGR5 led to the formation of extremely elongated filopodia (also called cytonemes) in HEK293 cells, suggesting robust, RSPO-independent activity in cytoskeleton reorganization as HEK293 cells express little RSPO endogenously (17). Just recently, it was shown that knock-in of endocytosis-deficient LGR5 into crypt stem cells resulted in decreased fitness of the stem cells without affecting proliferation (18). Intriguingly, LGR5 was reported to be constitutively coupled to the Gα_12/13_ subclass of heterotrimeric G proteins in the absence of RSPOs to activate the Rho GTPase pathway (19). This coupling, however, was neither verified independently nor evaluated in LGR5's role in the regulation of actin cytoskeleton. Taken together, these findings suggest that LGR5 has an important role in stem cell fitness through the control of the actin cytoskeleton and cell adhesion, although the exact mechanisms are yet to be defined.

The mammalian IQ motif-containing GTPase-activating proteins (IQGAPs) are a group of three related intracellular proteins (IQGAP1–3) with pivotal roles in the regulation of cytoskeletal structure, cell–cell adhesion, polarization, and migration (20–22). IQGAPs integrate signaling cross-talk within the cell through binding to and modulating the activities of a plethora of signaling molecules, including members of the Wnt pathway (APC, β-catenin, and E-cadherin), F-actin, mitogen-activated protein kinases, and Rho GTPases (20, 22). The Rho family of small GTPases consists of the Rac, Rho, and CDC42 subfamilies that are guanine nucleotide-binding proteins that cycle between an active GTP-bound state and an inactive GDP-bound form to regulate multiple actin dynamics and signal transduction pathways (23). Although IQGAP1 contains a GTPase-activating protein-like domain, it actually stabilizes GTP binding rather than catalyzes hydrolysis of GTP in Rac1/CDC42 (20–22). Instead, IQGAP1 binds to the active forms of Rac1 and CDC42 to coordinate actin assembly and control cell–cell adhesion (20, 22). Specifically, binding of Rac1/CDC42 to IQGAP1 inhibits the IQGAP1–β-catenin interaction, leading to an increase in membrane-bound β-catenin, cell–cell adhesion, and F-actin cross-linking (20, 24). Rac1/CDC42 binding to IQGAP1 is inhibited by phosphorylation at two serine sites (Ser-1441 and Ser-1443) of IQGAP1 (25–27). Here, we show that LGR5 interacts with IQGAP1 and decreases phosphorylation at these two serine sites, leading to increased binding of Rac1 and consequently enhancement of cell–cell adhesion. Ablation of LGR5 in colon cancer cells and in intestinal crypt stem cells results in a disorganized cytoskeletal structure with loss of cortical F-actin, suggesting an intricate role for the receptor in cancer and for retaining stem cells within the intestinal stem cell niche.

## Results

### Knock-out of LGR5 disrupts the cytoskeletal architecture of intestinal crypt organoids

**Figure 1.**
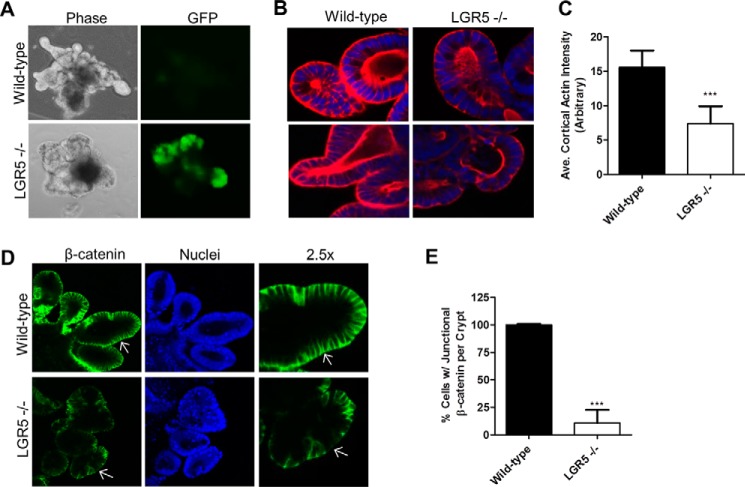
**Loss of LGR5 in intestinal crypt organoids resulted in disorganization of F-actin and β-catenin.** *A*, bright-field micrographs of wild-type and LGR5 KO mouse intestinal crypt organoids cultured in Matrigel. *B*, confocal microscopy images of F-actin (*red*) in wild-type and LGR5 KO organoids. Nuclei were counterstained using TO-PRO-3 (*blue*). *C*, quantification of cortical F-actin. *D*, confocal images of β-catenin in WT and LGR5 KO organoids. Nuclei were counterstained using TO-PRO-3 (*blue*). *Arrows* are included for image reference. *E*, quantification of β-catenin. *Error bars* are S.D. (*n* = 15–20 crypts). ***, *p* < 0.001 *versus* WT.

LGR5 expression is restricted to the crypt stem cells in the intestine, but its conditional knock-out was found to have no obvious effect on the proliferation and differentiation of the stem cells both *in vivo* and *ex vivo* (6, 28). We asked whether loss of LGR5 affects cytoskeletal structures of crypt stem cells given the effect of LGR5 on the actin cytoskeleton in cancer cell lines (15, 16). Intestinal organoids were generated from LGR5^−/−^ (LGR5-EGFP-IRES-creERT2) homozygotes and their wild-type (WT) littermates and cultured in Matrigel as described (29). LGR5 KO was verified by genotyping and positive GFP expression in the stem cells (Fig. 1*A*). The organoids were passaged two to three times, and no obvious difference was observed between organoids of LGR5^−/−^ and those of WT in growth and morphology. F-actin staining with rhodamine-phalloidin showed that LGR5^−/−^ organoids had significantly lower levels of cortical F-actin, particularly on the basolateral side of the crypts (Fig. 1, *B* and *C*). Additionally, β-catenin was disorganized in LGR5^−/−^ organoids (Fig. 1, *D* and *E*). In WT organoids, β-catenin was clearly localized to the cytoplasmic membrane, especially at the cell–cell junctions, with some cytoplasmic expression (Fig. 1*D*). Quantification of the staining indicated that only ∼11% of cells in LGR5^−/−^ crypts retained wild-type levels of β-catenin at the cell–cell junction (*p* ≤ 0.001) (Fig. 1*E*). These results suggest that LGR5 is critical for the organization of actin cytoskeletal structures in crypt stem cells and potentially cell–cell adhesion, but the exact mechanisms involved remain unknown.

### Overexpression of LGR5 alters actin cytoskeleton and increases cell–cell adhesion

**Figure 2.**
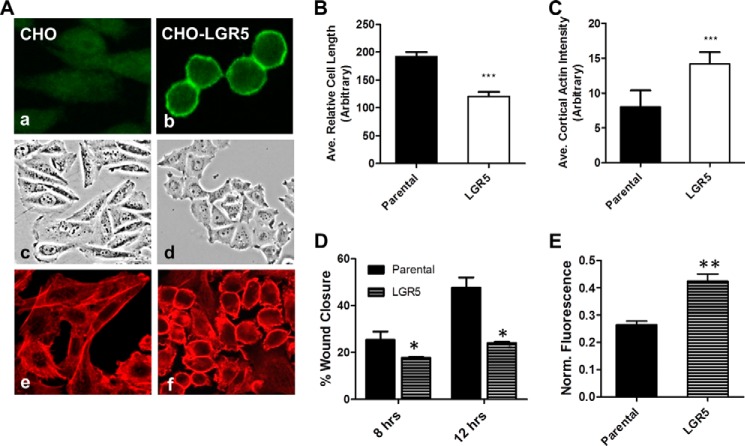
**LGR5 overexpression in CHO cells led to increase in cortical F-actin and cell–cell adhesion.** *A*, representative confocal images of ICC showing detection of LGR5 on the cell membrane at 4 °C in CHO-LGR5 stable cell line but not in parental CHO cells (*panels a* and *b*), phase-contrast images depicting morphological changes resulting from LGR5 overexpression (*panels c* and *d*), and confocal images of F-actin staining with rhodamine-phalloidin (*panels e* and *f*). *B* and *C*, quantification of cell length (*B*) and cortical actin (*C*) of CHO and CHO-LGR5 cells. *Error bars* are S.D. (*n* = 20–30 cells). ***, *p* < 0.001 *versus* parental CHO cells. *D*, wound healing assay results of CHO and CHO-LGR5 cells at 8 and 12 h postwounding. *Error bars* are S.E. (*n* = 3). *, *p* < 0.05 *versus* control CHO cells. *E*, results of cell–cell adhesion analysis using the calcein-AM assay. *Error bars* are S.E. (*n* = 3). **, *p* < 0.01 *versus* CHO cells.

To understand how LGR5 regulates actin cytoskeleton and cell adhesion, we examined the effect of overexpressing LGR5 in epithelial cell lines. CHO cells stably overexpressing full-length human LGR5 were obtained, and receptor expression was analyzed using LGR5-specific antibody. Immunocytochemistry (ICC) analysis showed that LGR5 was located on the cell surface (Fig. 2*A*, *panels a* and *b*). Interestingly, it was apparent that cells overexpressing LGR5 were slightly more rounded and substantially reduced in size, and they appeared more adherent and compact (Fig. 2*A*, *panels c* and *d*). These observations paralleled the morphological changes observed in cancer cell lines following LGR5 overexpression (15, 16). Quantification of relative cell length showed that CHO-LGR5 cells were shorter when compared with parental CHO cells (Fig. 2*B*). Phalloidin staining showed an apparent increase in cortical F-actin in CHO-LGR5 cells when compared with parental cells (Fig. 2*A*, *panels e* and *f*). Quantification of the average cortical actin fluorescence intensity per cell indicated a significant increase in intensity in CHO-LGR5 cells (*p* ≤ 0.001) (Fig. 2*C*). We then asked whether these LGR5-induced changes involve RSPO ligands. RT-PCR analysis showed that parental and CHO-LGR5 cell lines did not express any of the four RSPOs (supplemental Fig. S1), indicating that LGR5 has constitutive or RSPO-independent activity in regulating the actin cytoskeleton and cell morphology.

Given the changes induced by LGR5 in the actin cytoskeleton, the effects of LGR5 on cell migration and adhesion were also determined. CHO-LGR5 cells showed a significant reduction in cell migration using the wound healing assay (Fig. 2*D*). In the calcein-AM-based cell–cell adhesion assay, which measures the retention of a fluorescent dye in cells attached to other cells without the dye (30), CHO-LGR5 cells exhibited significantly stronger adhesion compared with parental CHO cells (Fig. 2*E*).

**Figure 3.**
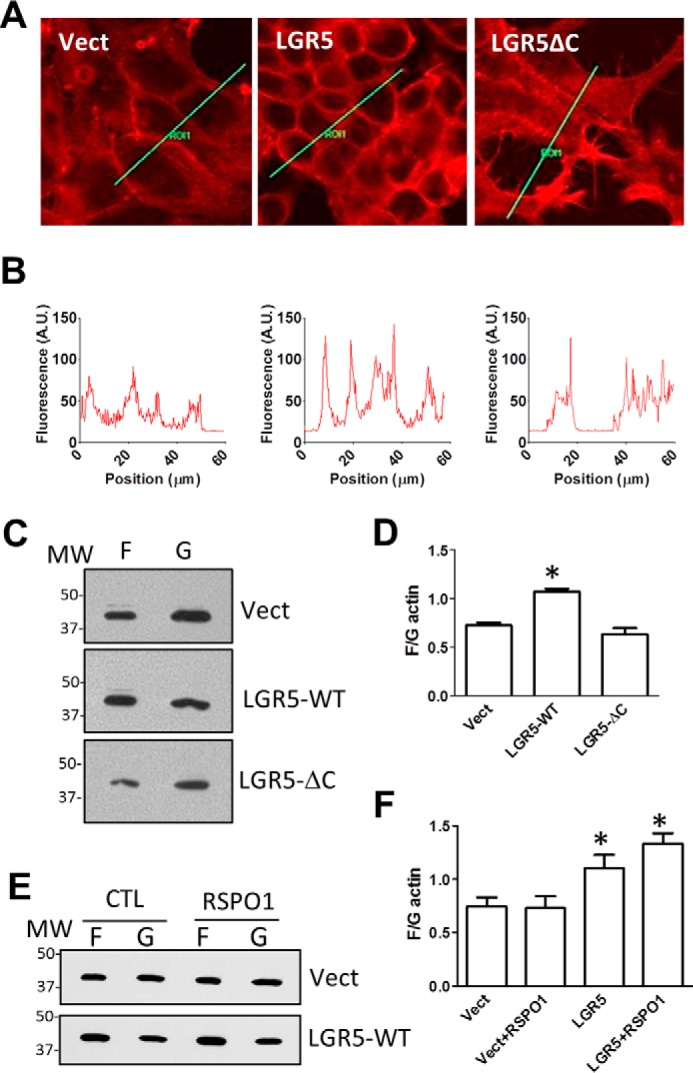
**Overexpression of LGR5 in HEK293T cells increased cortical F-actin levels.** *A*, confocal images of phalloidin staining of HEK293T cells expressing vector control, Myc-tagged LGR5-WT, or LGR5-ΔC. *B*, quantification of fluorescence of the lines drawn in *A. C*, representative WB results of G- and F-actin as separated by the Triton X-100 method. *D*, quantification of F- and G-actin WB results expressed as the ratio of F- *versus* G-actin. *E*, representative WB results of G- and F-actin of HEK293-vector or -LGR5-WT cells after treatment with RSPO1 (100 ng/ml) or vehicle overnight. *F*, quantification of WB results of G/F-actin. All *error bars* are S.E. (*n* = 3). *, *p* < 0.05 compared with vector (*Vect*) cells. *A.U.*, arbitrary units; *CTL*, control.

Previously, we reported the generation and characterization of HEK293T cells stably expressing vector, LGR5-WT, and LGR5-ΔC (endocytosis-impaired due to the deletion of the C-terminal tail sequence AA 837–907) in the regulation of Wnt/β-catenin signaling (31). Our findings showed that the C-terminal tail of LGR5 is not only important for internalization but also critical for regulating receptor signaling. Snyder *et al.* (32) reported that overexpression of an endocytosis-impaired LGR5 mutant with a truncated C-terminal tail led to formation of cytonemes in HEK293 cells, whereas LGR5-WT displayed few or no such cellular protrusions. Furthermore, the same LGR5 mutant was recently shown to reduce stem cell fitness *in vivo* by lineage tracing (18). Here, we examined the effect of Myc-tagged LGR5-WT and -ΔC overexpression on the actin cytoskeleton and cell adhesion. F-actin staining showed that cells overexpressing LGR5 displayed a more compact structure and increased levels of F-actin at cell–cell contacts (Fig. 3*A*), which were confirmed by quantification (Fig. 3*B*). Cells expressing LGR5-ΔC, however, displayed cytoneme-like structures without increased levels of F-actin at the cell–cell contacts (Fig. 3, *A* and *B*), consistent with the findings of Snyder *et al.* (32). F-actin and G-actin were then extracted from the three cell lines, and their relative levels were determined by immunoblot analysis and quantified (Fig. 3, *C* and *D*). Cells overexpressing LGR5-WT showed a higher ratio of F- to G-actin when compared with vector and LGR5-ΔC cells. Of note, HEK293 cells express RSPOs at very low levels as shown by RT-quantitative PCR (17) and are highly sensitive to RSPO stimulation in Wnt/β-catenin signaling (5, 6, 8), suggesting that LGR5-induced actin cytoskeleton changes are RSPO-independent. Indeed, treatment with RSPO1 overnight had no significant effect on the F- to G-actin ratio either in control or LGR5 cells (Fig. 3, *E* and *F*), confirming that the activity is RSPO-independent. Overall, these effects of LGR5 on F-actin structure and cell adhesion in CHO and HEK293 cells are similar to the phenotypes observed with overexpression of LGR5 in colon and liver cancer cell lines (15, 16). Importantly, the effects of LGR5 on the actin cytoskeleton were all displayed in the absence of RSPO stimulation (either endogenous or exogenous), indicating that these activities do not involve the two E3 ligases RNF43 and ZNRF3.

### LGR5 is not coupled to heterotrimeric G proteins

**Figure 4.**
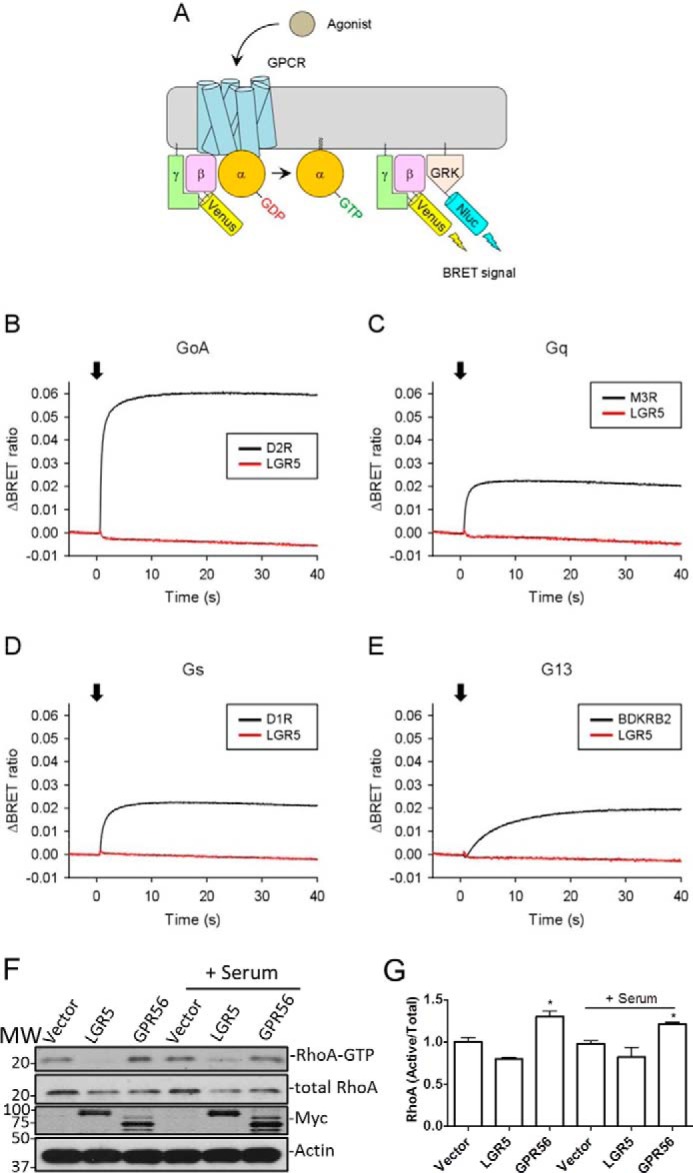
**LGR5 did not exhibit GEF activity with any of the four heterotrimeric G protein subfamilies.** *A*, a schematic diagram showing the principle of the BRET assay system. Agonist-bound GPCR exerts GEF activity, leading to the dissociation of inactive heterotrimeric G proteins into active GTP-bound Gα and Venus-Gβγ subunits. The free Venus-Gβγ then interacts with masGRK3ct-Nluc to produce the BRET signal. *B–E*, real-time measurement of GEF activity in living cells. HEK293T/17 cells were transfected with GPCR (dopamine D2 (*D2R*), M3 acetylcholine (*M3R*), dopamine D1 (*D1R*), or bradykinin B2 receptor (*BDKRB2*), or LGR5) and Gα subunit (Gα_oA_, Gα_q_, Gα_s_, or Gα_13_) with the BRET sensor pair Venus-Gβγ and masGRK3ct-Nluc. Agonists (100 nm dopamine for dopamine D1 and D2 receptors, 100 nm acetylcholine for M3 acetylcholine receptor, 100 nm bradykinin for bradykinin B2 receptor, and 20 nm RSPO3 for LGR4 and LGR5) were applied on transfected cells to stimulate GPCRs at 0 s. *F*, representative WB results of active RhoA GTPase pulldown assay in stable HEK293T cells overexpressing vector, Myc-LGR5, or Myc-GPR56. Cells were starved overnight and then treated ±10% serum for15 min. *G*, quantification of WB results of Rho GTPase. *Error bars* are S.E. (*n* = 2). *, *p* < 0.05 compared with vector and LGR5 cells.

Next, we attempted to identify the intracellular mechanism that mediates the effect of LGR5 on the actin cytoskeleton and cell adhesion. Previously, we and others showed that LGR5 is not coupled to any of the three types of heterotrimeric G proteins that give rise to secondary messenger formation (cAMP or Ca^2+^) or to β-arrestin in response to RSPO stimulation (5, 6, 8). Intriguingly, Kwon *et al.* (19) reported that LGR5 coupled to the Gα_12/13_–Rho GTPase pathway to activate the serum response factor response element pathway in the absence of RSPO stimulation. However, neither binding nor direct activation of Gα_12/13_ (exchange of GDP for GTP) by LGR5 was demonstrated (19). As the Gα_12/13_ pathway plays a critical role in the control of actin dynamics and cell migration, we examined whether LGR5 activates Gα_12/13_ or any of the other heterotrimeric G protein subclasses using a direct method. Activation of heterotrimeric G proteins by 7TM receptors can be monitored directly by highly sensitive assays based on changes in bioluminescence resonance energy transfer (BRET; Fig. 4*A*) that are dependent on the release of the Gβγ subunit from the activated GPCR and its subsequent binding to a fragment of GRK3 (33–35). Using this assay, we tested whether LGR5 increases guanine nucleotide exchange factor (GEF) activity of any of the four major subtypes of heterotrimeric G proteins (Gα_s_, Gα_i/o_, Gα_q_, and Gα_12/13_) with RSPO stimulation. No significant signal by LGR5 was detected with any of the four G proteins that were tested (Fig. 4, *B–E*), whereas all positive control receptors showed robust activity (Fig. 4, *B–E*). We then performed a GTPase pulldown assay to measure activation of RhoA (which occurs downstream of Gα_12/13_) in HEK293 cell lines stably overexpressing vector, LGR5, or GPR56, a GPCR known to activate the Gα_12/13_/RhoA signaling pathway constitutively (36). Our results showed that GPR56 increased active RhoA levels with or without serum stimulation compared with vector cells, whereas LGR5 caused a slight decrease (not significant) in the level of Rho-GTP (Fig. 4, *F* and *G*). Based on these results, we conclude that LGR5 does not appear to bind to or function through Gα_12/13_ and further confirmed that LGR5 is not coupled to the other three subclasses of heterotrimeric G proteins.

### LGR5 interacts with IQGAP1

**Figure 5.**
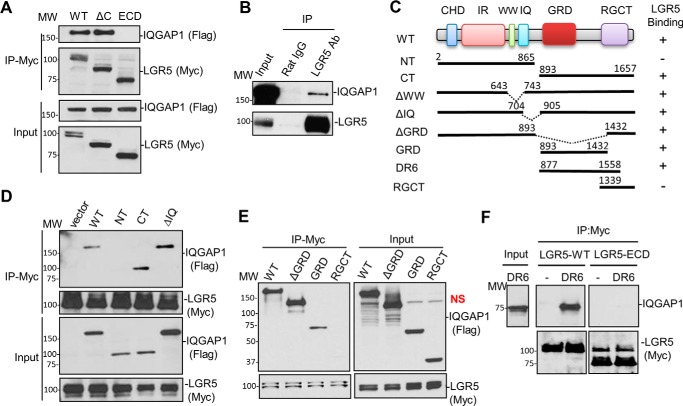
**LGR5 binds to the C-terminal half of IQGAP1.** *A*, WB results of FLAG-tagged IQGAP1 co-IP with Myc-LGR5-WT, Myc-LGR5-ΔC, and membrane-tethered Myc-LGR5-ECD. *B*, WB results of co-IP of endogenous IQGAP1 and LGR5 in LoVo cells. *C*, a schematic diagram of IQGAP1 domain structure and the LGR5 binding results of various mutants tested. The domains are as follows: *CHD*, calponin homology domain; *IR*, IQGAP-specific repeat motif; *WW*, domain with two conserved Trp (W) residues; *IQ*, calmodulin-binding IQ motif; *RGCT*, RasGAP C terminus; *NT*, N terminus; *CT*, C terminus. The *numbers* denote the amino acid residues where mutant proteins/deletion regions start and end. *D*, WB results of co-IP of FLAG-IQGAP1 mutants with LGR5-WT using whole-cell lysates. *E*, WB results of co-IP of FLAG-IQGAP1 C-terminal tail mutants with Myc-LGR5-WT using whole-cell lysates. *F*, WB results of co-IP of purified IQGAP1 fragment (DR6; amino acids 877–1558) with LGR5-WT. *NS*, indicates nonspecific band. Each experiment was repeated two to three times, and shown here are representative WB results.

LGR4 was found to interact with the intracellular scaffold protein IQGAP1 to potentiate Wnt signaling, and it regulates focal adhesion formation and cell migration (11). IQGAP1 plays a major role in the control of the actin cytoskeleton and cell adhesion and migration, largely through modulation of the small G protein Rac1 and CDC42 (37, 38). Given the homology of LGR4 and LGR5 and that IQGAP1 and IQGAP3 appeared as proteins that co-purified with both receptors in mass spectrometry analysis (6), we tested whether LGR5 also interacts with IQGAP1. Using recombinant overexpression and co-IP analysis in HEK293T cells, we found that FLAG-IQGAP1 did interact with Myc-tagged LGR5-WT as well as with the C-terminal tail-truncated mutant LGR5-ΔC (31) (Fig. 5*A*). As a negative control, we show that IQGAP1 was not pulled down with Myc-tagged LGR5-ECD anchored to the membrane with a single transmembrane domain (Fig. 5*A*). These results indicate that IQGAP1 binds to the 7TM domain of LGR5, and this interaction does not require the C-terminal tail (AA 837–907). To verify that this interaction is not due to overexpression, co-IP analysis was carried out with the LoVo colon cancer cell line, which has high endogenous levels of LGR5 (39). Indeed, endogenous IQGAP1 in LoVo cells was pulled down using an LGR5-specific antibody (Fig. 5*B*). To map the IQGAP1 domains that interact with LGR5, we utilized a series of FLAG-IQGAP1 truncation and deletion mutants (Fig. 5*C*). Co-IP studies demonstrated that LGR5 binds to the C-terminal half of IQGAP1 spanning amino acids 893–1657 (Fig. 5, *C–E*). Unlike LGR4 (11), however, we detected weak binding of LGR5 to the GRD domain but strong binding to a variant lacking the GRD domain (ΔGRD) (Fig. 5, *D* and *E*). We then tested whether LGR5 could bind to a purified IQGAP1 fragment spanning amino acids 877–1558 (DR6 or GRDD1-RGCT) that was expressed in *Escherichia coli*, purified, and shown to bind to CDC42 (26). LGR5-WT, but not LGR5-ECD, pulled down DR6 (Fig. 5*F*). Overall, these results suggest that the 7TM domain of LGR5 interacts with the C-terminal portion of IQGAP1, although the exact motifs from each partner have yet to be mapped.

### LGR5 decreases phosphorylation of IQGAP1 to enhance IQGAP1–Rac1 interaction

**Figure 6.**
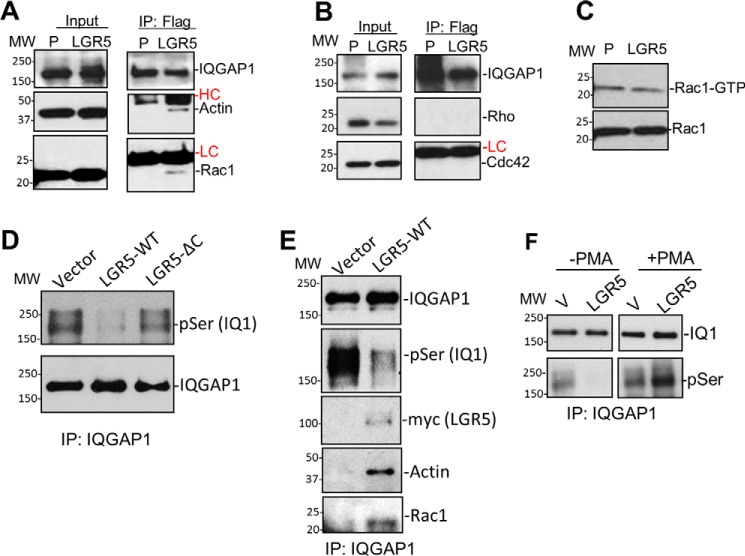
**LGR5 increased binding of Rac1 to IQGAP1 via regulating phosphorylation of IQGAP1 at Ser-1441/1443.** *A*, WB results of IQGAP1, Rac1, and actin following IP of IQGAP1 in parental CHO (*P*) and CHO-LGR5 cells. *HC* and *LC*, heavy and light chains of the IP antibody, respectively. *B*, WB results of IQGAP1, Rho, and CDC42 following IP of IQGAP1. *C*, WB of active (GTP-bound; binding to PAK1-PBD) and total Rac1. *D*, WB results of Ser(P) of IQGAP1 (*pSer (IQ1)*) probed by anti-phosphoserine antibody following IP of IQGAP1 in HEK293 cells overexpressing LGR5 or LGR5-ΔC (lacking C-terminal tail). *E*, WB results of Ser(P), LGR5, actin, and Rac1 following IP of IQGAP1. *F*, WB results of Ser(P) of IQGAP1 in HEK293-vector (*V*) or -LGR5 cells before and after treatment with PMA. Each experiment was repeated two to three times, and shown here are representative WB results.

IQGAP1 regulates the actin cytoskeleton by functioning as an effector of CDC42 or Rac1 (37). We examined whether LGR5 affects the binding of IQGAP1 to CDC42, Rac1, or both. Using co-IP analysis, we found that Rac1, but not CDC42 or RhoA, was pulled down with IQGAP1 in CHO-LGR5 cells (Fig. 6, *A* and *B*). In contrast, we did not detect any of the Rho GTPases in association with IQGAP1 in control CHO cells under the same conditions (Fig. 6, *A* and *B*). Furthermore, actin consistently co-precipitated with IQGAP1 in CHO-LGR5 cells but was not detectable in parental CHO cells (Fig. 6*A*). We then tested whether the levels of total “free” active Rac1 (*i.e.* not bound to IQGAP1) were altered due to LGR5 overexpression using a GST-PBD (PAK1) pulldown assay. Of note, IQGAP1 binds active GTPases with higher affinity and different specificity than PAK1 PBD (40). The PBD-bound active Rac1 levels were equivalent for each cell line (Fig. 6*C*), suggesting that LGR5 may stabilize the binding of Rac1 to IQGAP1 without affecting the overall levels of free active Rac1. In addition, LGR5-mediated binding of Rac1/actin to IQGAP1 was not affected by RSPO treatment (supplemental Fig. S2A), again suggesting that the effect of LGR5 on the actin cytoskeleton is independent of RSPO stimulation.

Binding of Rac1/CDC42 to IQGAP1 is regulated by phosphorylation of IQGAP1 at two sites (Ser-1441 and -1443) (25, 26, 41). Detailed kinetic analysis showed that phosphorylation at Ser-1443 led to a decrease in the affinity of human IQGAP1 for Rac1 and CDC42 (27). To test whether LGR5 regulates IQGAP1–Rac1 interaction through modulation of IQGAP1 phosphorylation, we probed the level of phospho-IQGAP1 with an anti-phosphoserine antibody (no antibody specifically against Ser-1441 or Ser-1443 of IQGAP1 is available (42)). Overexpression of LGR5-WT in HEK293 cells led to a drastic decrease in the level of phospho-Ser (Ser(P)) of IQGAP1 when compared with vector cells (Fig. 6*D*). Remarkably, LGR5-ΔC, which does not increase cortical F-actin, did not alter the levels of Ser(P) of IQGAP1 (Fig. 6*D*). Follow-up co-IP analysis confirmed that IQGAP1 showed increased binding to actin and Rac1 in HEK293 cells (Fig. 6*E*). To examine whether the decreased level of Ser(P) of IQGAP1 in LGR5 cells is due to inhibition of phosphorylation or enhancement of dephosphorylation, LGR5 and vector cells were treated with PMA for 10 min, which induces IQGAP1 phosphorylation at Ser-1441/1443, and the Ser(P) levels of IQGAP1 were compared. As shown in Fig. 6*F*, no difference was seen between LGR5 and vector in response to PMA treatment, suggesting that LGR5 did not inhibit acute phosphorylation of IQGAP1 by PKC. To confirm that the detected phospho-IQGAP1 signal was specific for Ser-1441/1443 phosphorylation, we generated an IQGAP1 mutant with both serines changed to alanine (SKS → AKA). As shown by IP in supplemental Fig. S2B, no signal was detected with the mutant IQGAP1, whereas IQGAP1-WT showed a strong signal following PMA treatment. These results suggest that the LGR5-induced decrease in IQGAP1 phosphorylation at Ser-1441/1443 was probably due to increased dephosphorylation.

### Knockdown of LGR5 in colon cancer cells leads to loss of IQGAP1-associated Rac1/actin and disruption of cytoskeletal structure

**Figure 7.**
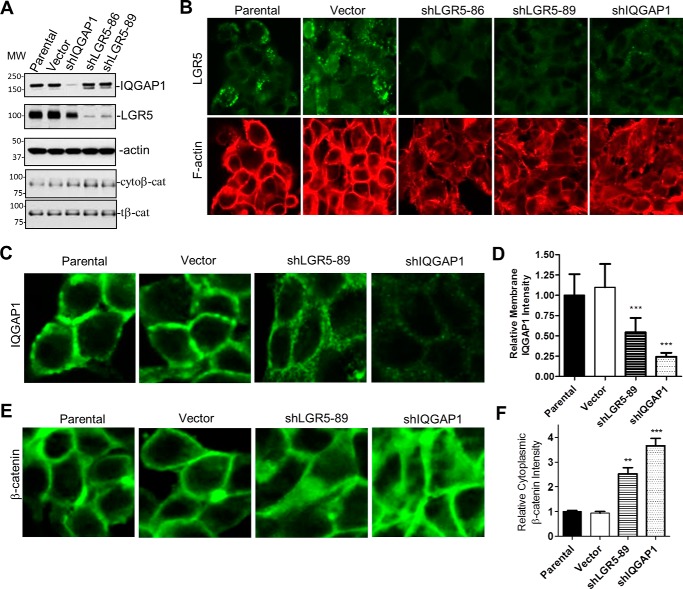
**KD of LGR5 in LoVo colon cancer cells altered cytoskeletal structure and decreased the level of membrane-associated IQGAP1 and β-catenin.** *A*, WB results in control and LoVo cells with KD of IQGAP1 or LGR5. *B*, confocal images of LGR5 (*green*) in control and cells with KD of IQGAP1 or LGR5. F-actin was stained with rhodamine-labeled phalloidin (*red*). *C*, confocal images of IQGAP1 in control and LoVo cells with KD of LGR5 or IQGAP1. *D*, quantification of membrane-associated IQGAP1. *Error bars* are S.E. (*n* = 20–30 cells). ***, *p* ≤ 0.001 *versus* parental and vector cells. *E*, confocal images of β-catenin. *F*, quantification of cytoplasmic β-catenin based on relative signal intensity. *Error bars* are S.E. (*n* = 20–30 cells). ** and ***, *p* ≤ 0.01 and 0.001, respectively, compared with parental and vector cells. Images in *C* and *E* are 2.5× *magnification* compared with *B. cyto*β*-cat*, cytoplasmic β-catenin; *t*β*-cat*, total β-catenin.

The LoVo colon cancer cell line is mutated in the tumor suppressor and Wnt inhibitor APC and expresses high levels of LGR5 and IQGAP1 (39). Microarray data of this cell line in the Cancer Cell Line Encyclopedia showed no expression of RSPOs (43), which we confirmed by RT-quantitative PCR analysis. LoVo cell lines stably expressing two independent LGR5 shRNA constructs, shLGR5-86 and shLGR5-89, along with vector control were generated. Both shRNA constructs yielded a ≥90% reduction in LGR5 as verified by WB and ICC (Fig. 7, *A* and *B*, *top panel*). A LoVo cell line stably expressing an IQGAP1 shRNA (shIQGAP1-85), which we used previously in lung cancer cell lines (11), was also generated, and loss of IQGAP1 was confirmed by WB (Fig. 7*A*).

We first examined F-actin structures in these cell lines and found that, strikingly, shIQGAP1, shLGR5-86, and shLGR5-89 cell lines all displayed a significant loss of cortical F-actin and showed an overall disorganized cytoskeletal structure when compared with control cell lines (Fig. 7*B*, *lower panels*). Correspondingly, loss of LGR5 expression resulted in reduced levels of IQGAP1 localized to the plasma membrane at cell–cell adhesion sites (Fig. 7, *C* and *D*). Using ICC, we also analyzed E-cadherin and β-catenin, which are critical to the formation and stabilization of cell–cell adhesion (24). Loss of LGR5 or IQGAP1 resulted in decreased membrane-associated β-catenin accompanied by increased β-catenin levels in the cytoplasm (Fig. 7, *E* and *F*), which were also confirmed by WB (Fig. 7*A*, *lower panel*). However, we did not observe any obvious changes in the level of E-cadherin or its localization (supplemental Fig. S3). These results suggest that LGR5–IQGAP1 functions to regulate β-catenin localization and is essential for the formation and/or stabilization of cortical F-actin.

**Figure 8.**
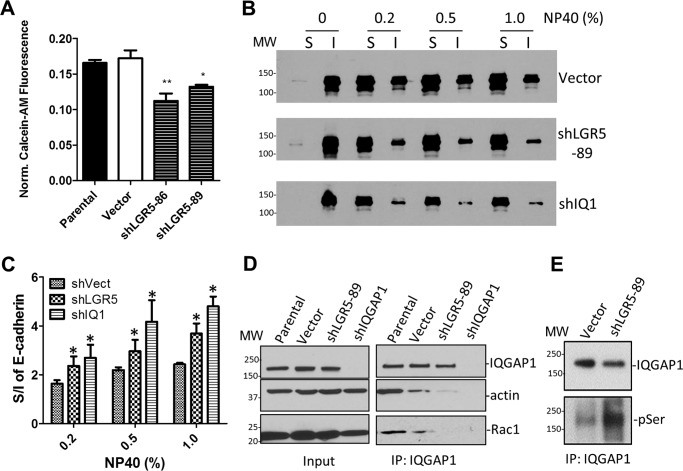
**KD of LGR5 in LoVo cells resulted in loss of IQGAP1 interaction with Rac1/actin and decreased cell–cell adhesion.** *A*, results of calcein-AM cell–cell adhesion assay. *Error bars* are S.E. (*n* = 3). *, *p* < 0.05 and **, *p* ≤ 0.01 *versus* controls. *B*, representative WB analysis results of soluble (*S*) and insoluble (*I*) E-cadherin in LoVo cells with vector control, KD of LGR5 (shLGR5-89), or KD of IQGAP1 (*shIQ1*) when extracted with the indicated concentrations of Nonidet P-40 (*NP40*). *C*, quantification of WB results of E-cadherin. *Error bars* are S.E. (*n* = 3). *, *p* < 0.05 compared with vector cells. *D*, WB results of IQGAP1, actin, and Rac1 in control and LoVo cells with KD of LGR5 or IQGAP1. *E*, WB of serine phosphorylation of IQGAP1 in LGR5 KD LoVo cells.

Formation of stable E-cadherin–mediated cell–cell adhesion depends on cortical F-actin, which is connected to the adhesion complex via α-catenin (44). The loss of cortical F-actin in LGR5 and IQGAP1 KD cells suggests that loss of LGR5–IQGAP1 interaction may decrease cell–cell adhesion. Using the calcein-AM fluorescence-based cell–cell adhesion assay, we demonstrated that KD of LGR5 resulted in a reduction in cell–cell adhesion (Fig. 8*A*). Cells with poorly organized cortical F-actin are also expected to have increased levels of soluble E-cadherin (45). Indeed, we found that LoVo cells lacking LGR5 showed a significant increase in the ratio of soluble *versus* insoluble E-cadherin when extracted by Nonidet P-40 (Fig. 8, *B* and *C*).

As overexpression of LGR5-WT led to a decrease in phosphorylation of Ser-1441/1443 of IQGAP1 and consequently increased Rac1 binding to IQGAP1, we tested whether the opposite was true in LoVo cells with KD of LGR5. Co-IP was performed with an anti-IQGAP1 antibody to pull down endogenous IQGAP1 in parental, vector, and shLGR5-89 cells (shIQGAP1 cells were included as a negative control for IQGAP1 antibody specificity) (Fig. 8*D*). As expected, we found that KD of LGR5 resulted in loss of Rac1 and actin association with IQGAP1 (Fig. 8*D*). Furthermore, the level of IQGAP1 phosphorylation was found to be higher in LGR5 KD cells (Fig. 8*E*). Together with the LGR5 overexpression data, these findings coherently suggest a mechanism whereby LGR5 reduces phosphorylation of IQGAP1 at Ser-1441/1443 to increase the IQGAP1–Rac1 interaction and ultimately enhance cell–cell adhesion via regulating the actin cytoskeleton.

## Discussion

LGR5 is currently the most widely recognized and utilized stem cell marker in the gastrointestinal tract and several other epithelial tissues, and it is up-regulated in a substantial fraction of solid tumors (1, 2). However, the exact roles and mechanisms of LGR5 in normal adult stem cells and cancer cells remain poorly defined. Notably, expression of LGR5 is not essential for the survival of crypt stem cells *in vivo* or *ex vivo* (6, 28). The function of LGR5 in cancer cells appeared to be tumor-type dependent with tumor suppressor-like activity in colon and liver cancer cells and tumor-promoting activity in other cancer cell types (15, 16, 46). Mechanistically, multiple studies showed that LGR5 can bind the RSPO1–4 family of stem cell factors to potentiate Wnt/β-catenin signaling in HEK293T cells (5, 6, 8). RSPOs (with the exception of RSPO4) also bind to the two E3 ligases RNF43 and ZNFR3, which ubiquitinate Wnt receptors for degradation, and the co-crystal structure of LGR5–RNF43–RSPO has been solved (47, 48). Based on these studies, the current model is that LGR5, like LGR4, functions as a high-affinity co-receptor of RSPOs, facilitating the binding of RSPOs to inhibit RNF3/ZNRF3, leading to enhanced Wnt signaling (49). However, direct evidence of RSPO–LGR5 mediating the inhibition of RNF43/ZNRF3 has never been reported. Furthermore, KO of LGR5 in multiple tissues led to increased levels of Axin2 (marker of Wnt/β-catenin signaling), suggesting that LGR5 does not play a major, positive role in Wnt/β-catenin signaling in stem cells *in vivo* (12–14). Remarkably, a consistent finding is that LGR5 overexpression in cancer cells alters the actin cytoskeleton structure and increases cell–cell adhesion in the absence of endogenous or exogenous RSPO stimulation (15, 16). Such LGR5-induced changes in the actin cytoskeleton are unlikely to be mediated by inhibition of RNF43/ZNRF3 because these changes occur in the absence of RSPOs, which are essential for interacting with RNF43/ZNRF3. Here, we present evidence for a novel mechanism whereby LGR5 is coupled to the intracellular scaffold signaling protein IQGAP1 to regulate the actin cytoskeleton and cell–cell adhesion.

IQGAP1 interacts with a plethora of receptors and effectors to coordinate signaling and regulate actin cytoskeleton dynamics. We found that LGR4 interacts with the GRD domain of IQGAP1 to potentiate both the canonical and non-canonical pathways of Wnt signaling (11). In comparison, LGR5 interacts not only with the GRD domain but also with a C-terminal domain of IQGAP1. Interestingly, overexpression of LGR4 and LGR5 has an opposite effect on the actin cytoskeleton and cell adhesion and migration (11, 15, 16, 50, 51). The distinctive domains of IQGAP1 involved in binding to LGR5 may underlie the differential effect of LGR4 and LGR5 on these cellular processes and morphology. The 7TM domains between LGR4 and LGR5 are highly conserved, whereas their C-terminal tails are quite divergent. LGR5 lacking its C-terminal tail is endocytosis-impaired, potentiates Wnt/β-catenin signaling (31, 52), and induces the formation of cytonemes in HEK293 similar to those induced by full-length LGR4 (32). These findings imply that the C-terminal tail of LGR5, although not essential for IQGAP1 binding, may interact with additional domains on IQGAP1 or other proteins bound to IQGAP1 to exert effects distinct from those of LGR4. As LGR5 does not appear to affect acute phosphorylation of IQGAP1, a potential mechanism is that the C-terminal tail of LGR5 recruits a Ser/Thr phosphatase to dephosphorylate IQGAP1.

**Figure 9.**
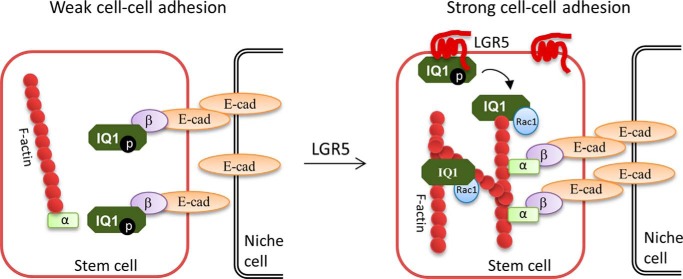
**A schematic diagram illustrating the mechanism of LGR5 in the regulation of cell–cell adhesion through the IQGAP1–Rac1 pathway.** In the absence of LGR5 (*left panel*), phosphorylated IQGAP1 (*IQ1*) is free of Rac1-GTP and can thus bind to β-catenin (β) to disrupt the interaction of β-catenin and α-catenin (α), leading to separation of the E-cadherin (*E-cad*) adhesion complex from the actin cytoskeleton and weak cell–cell adhesion. In the presence of LGR5 (*right panel*), the receptor reduces phosphorylation of IQGAP1, which then binds Rac1-GTP, leading to loss of binding to β-catenin as well as enhanced cross-linking of F-actin. This results in the linkage of E-cadherin adhesion complex to the cytoskeleton and stronger cell–cell adhesion.

Initially, we found that overexpression of LGR5 consistently led to increased binding of Rac1 and actin to IQGAP1 in co-IP analysis. Because it was reported that binding of Rac1/CDC42 to IQGAP1 is inhibited by phosphorylation at Ser-1441/1443 of IQGAP1 (26, 27, 41), we asked whether LGR5 modulates phosphorylation of IQGAP1 at these sites to regulate IQGAP1–Rac1 interaction. Indeed, LGR5 overexpression and KD of LGR5 led to a significant decrease and increase in Ser-1441/1443 phosphorylation, respectively, and LGR5 had no effect on PMA-induced phosphorylation of IQGAP1. Furthermore, overexpression of LGR5-ΔC, which induced cytoneme formation without an increase in total F-actin, had no effect on phosphorylation (Fig. 5*D*). These results suggest that the C-terminal tail of LGR5 regulates phosphorylation of the two serine sites, potentially through recruitment of phosphatase(s). In turn, decreased levels of phosphorylated IQGAP1 at Ser-1441/1443 led to increased Rac1 binding to IQGAP1. Importantly, Rac1-bound IQGAP1 can no longer interact with β-catenin but can still bind to and cross-link F-actin filaments (20), leading to an increase in E-cadherin–β-catenin–α-catenin complex formation and F-actin cross-linking and therefore stronger cell–cell adhesion (20, 24). Thus, we propose a model whereby LGR5-induced depletion of IQGAP1 phosphorylation increases IQGAP1–Rac1 interaction with concomitant loss of IQGAP1–β-catenin interaction, eventually leading to strengthening of cell–cell adhesion (Fig. 9). Furthermore, the slight increase of Wnt/β-catenin signaling in LGR5 KO tissues (12–14) may be explained by release of β-catenin from adherens junction sites at the plasma membrane in LGR5 KO cells with weakened cell–cell adhesion (53).

The critical role of LGR5 in the control of cell–cell adhesion may also shed light on reasons why LGR5 is specifically expressed by adult stem cells in various epithelial tissues. Stem cell niches provide an adhesive milieu that can selectively retain daughter stem cells but not differentiated daughter cells (54). In the small intestine, LGR5^+^ stem cells (∼14 cells per crypt) are interspersed with Paneth cells throughout the base of the crypt or stem cell niche (55). *In vivo* imaging has shown that LGR5^+^ stem cells located at the crypt base experience a survival advantage over border stem cells that can be passively displaced into the transit-amplifying region along the sides of the crypt (56). It is thus tempting to speculate that the stem cells that form stronger cell–cell adhesion contacts have a competitive advantage in terms of being retained in the niche and remaining as stem cells. Given that LGR5 is not essential for the growth and survival of crypt stem cells, it is conceivable that the receptor may primarily function to promote strong cell–cell adhesion in stem cells, which in turn retains these cells within the crypt base to maintain homeostasis in the intestinal epithelium. Indeed, a recent study showed that intestinal stem cells expressing LGR5 without its C-terminal tail were less “fit” *in vivo* as manifested by their decreased lifespan in the intestine (18) This is consistent with our model that LGR5 lacking the C-terminal tail cannot reduce phosphorylation of IQGAP1, leading to a lower level of Rac1 binding and a decrease in cell–cell adhesion and thus faster elimination *in vivo*.

## Experimental procedures

### Plasmids and cloning

Plasmids encoding Myc-LGR5, Myc-LGR5-ECDTM, Myc-LGR5-ΔC, and all FLAG-tagged mouse IQGAP1 deletion/truncation mutants were generated as described previously (5, 11). Myc-tagged full-length human GPR56 was cloned into pIRESpuro3 using standard PCR-based methods. FLAG-tagged mouse IQGAP1-CT truncation mutant was constructed by amplifying the fragment containing AA 962–1657 from mouse IQGAP1 pCMV-sport6 (Addgene). The PCR product was subcloned into a pcDNA3.1 vector modified to incorporate an N-terminal FLAG tag. GST-PBD (PAK1) was a gift from Dr. Jeffrey Frost (University of Texas Health Science Center-Houston). The heterotrimeric G protein activation plasmids were described previously (33).

### Recombinant proteins, antibodies, and chemicals

Recombinant human RSPO1 was purchased from R&D Systems. IQGAP1 DR6 recombinant protein was produced and purified from *E. coli* as reported previously (26). For crypt organoid cultures, Noggin was purchased from Peprotech; *N*-acetylcysteine was from Sigma; and N2, B27, and mouse EGF were all purchased from Life Technologies. All commercial antibodies were used in accordance with the manufacturers' guidelines. For Western blot analysis, anti-LGR5 (Abcam ab75732), anti-Rac1 (BD Biosciences catalog number 610650), anti-IQGAP1 (BD Biosciences catalog number 610611), anti-FLAG (Sigma catalog number F7425), anti-Gα_13_ (Santa Cruz Biotechnology sc-410), and Cell Signaling Technology antibodies anti-CDC42 (catalog number 2466), anti-Rho (catalog number 8789), anti-Myc (catalog number 2272 or 2278), anti-β-catenin (catalog number 9562), anti-E-cadherin (catalog number 3195), anti-phospho-Ser PKC substrate antibody (catalog number 2261), and anti-β-actin (catalog number 4970) were used. Specificities of all antibodies were confirmed based on protein size and correlation with recombinant expression. For ICC experiments, anti-β-catenin-Alexa Fluor 488 (Cell Signaling Technology catalog number 2849), anti-E-cadherin-Alexa Fluor 488 (Cell Signaling Technology catalog number 3199), anti-IQGAP1 (Bethyl Laboratories catalog number A301), anti-LGR5 (BD Biosciences catalog number 562731), and anti-Myc-Cy3 (Sigma catalog number C6594) were used. TO-PRO-3, rhodamine-phalloidin, and rabbit anti-rat Alexa Fluor 488, and goat anti-rabbit Alexa Fluor 488 secondary antibodies were purchased from Life Technologies.

### Cell culture and shRNA stable cell line generation

HEK293T and CHO-K1 cells were purchased from ATCC. HEK293T and CHO-K1 cells were cultured in high-glucose DMEM and F-12/Ham's medium, respectively, and supplemented with 10% fetal bovine serum (FBS) and penicillin/streptomycin (pen/strep) at 37 °C with 95% humidity and 5% CO_2_. CHO-LGR5 cells were purchased from DiscoveRx and maintained in F-12/Ham's medium supplemented with 10% FBS, 300 μg/ml hygromycin, 800 μg/ml geneticin, and pen/strep. LoVo cells were obtained from the laboratory of Dr. Shao-Cong Sun at M. D. Anderson Cancer Center, Houston,TX, and maintained in RPMI 1640 medium with 10% FBS and pen/strep. To generate stable KD of LGR5 or IQGAP1, LoVo cells were infected with lentivirus particles produced by co-transfecting HEK293T cells with a pLKO.1 vector incorporating either the LGR5- or IQGAP1-targeted shRNA and packaging plasmids psPAX2 and pMD2.G using FuGENE 6 (Roche Applied Science). Virus-infected cells were selected with puromycin. The shRNA clones were from GE Dharmacon with clone numbers as follows: human LGR5, TRCN0000011586 (shLGR5-86), human LGR5, TRCN0000011589 (shLGR5-89), and human IQGAP1, TRCN0000047485 (shIQGAP1-85). The corresponding sequences can be accessed on the GE Dharmacon website.

### Intestinal crypt organoid culture

All animal experiments were performed in accordance with a protocol approved by the Animal Protocol Review Committee of the University of Texas Health Science Center at Houston. B6.129P2-LGR5tm1(cre/ERT2)Cle/J mice were purchased from The Jackson Laboratory and bred to produce wild-type and LGR5 KO mutant offspring. Because LGR5 KOs are perinatal lethal, pups were sacrificed, and intestines were collected immediately after birth. Tail genotyping was conducted following the standard PCR protocol provided by The Jackson Laboratory. Mouse intestinal crypt organoid cultures were established as published previously (29). Briefly, small intestines were harvested and washed to remove contaminants and villi. Intestinal fragments were incubated in EDTA for 30 min, strained, and pelleted. Crypts were resuspended in Matrigel with DMEM/F-12 containing 10 mm HEPES, GlutaMAX, 1× B27, 1× N2, 1 mm *N*-acetylcysteine, 50 ng/ml mouse EGF, 100 ng/ml mouse Noggin, 20 ng/ml RSPO1, and pen/strep. The medium was replenished every 2–3 days, and the crypt organoids were mechanically broken down with a glass pipette and passaged every 5–6 days.

### RT-PCR

Total RNA was extracted by lysing the cells with TRIzol (Life Technologies) followed by the successive addition of chloroform and isopropanol for phase separation and RNA precipitation, respectively. Samples were run through RNeasy Mini kit columns (Qiagen) according to the manufacturer's protocol, eluted with RNase-free water, and DNase-treated. cDNA was produced using the iScript kit (Bio-Rad).

### Western blotting and immunoprecipitation

Heterotrimeric G protein BRET assays were performed as described before (35). Rho-GTP pulldown was carried out using an assay kit (Cytoskeleton, Inc. catalog number BK036). F-actin and G-actin were separated and extracted using the Triton X-100 assay essential as described previously (57). Quantification of soluble *versus* insoluble E-cadherin was performed as described before (45). For WB, cells were lysed with radioimmune precipitation assay buffer (50 mm Tris-Cl, pH 7.4, 150 mm NaCl, 1 mm DTT, 1% Triton X-100, 1% sodium deoxycholate, 0.1% SDS) or GTPase Buffer (25 mm Tris, pH 7.5, 30 mm MgCl_2_, 0.13 m NaCl, 0.5% Nonidet P-40) for experiments involving GTPase activity. Both buffers were supplemented with protease and phosphatase inhibitors. HRP-labeled secondary antibodies (Cell Signaling Technology) were utilized for detection along with the standard ECL protocol. For co-immunoprecipitation experiments, cell lysates were incubated ∼3–4 h at 4 °C with anti-Myc magnetic beads (Cell Signaling Technology), anti-FLAG magnetic beads (Sigma), glutathione magnetic beads (Sigma), or primary antibody and protein A/G-agarose beads (Santa Cruz Biotechnology). Precipitates were washed with lysis buffer followed by PBS and boiled with 2× SDS sample buffer prior to loading for SDS-PAGE and Western blot analysis. G protein BRET assays were carried out as described previously (33). All ligand treatments were performed with Wnt3a-conditioned medium (diluted 1:5) and 30 ng/ml RSPO unless otherwise stated. All experiments were performed at least three times.

### Immunocytochemistry and confocal microscopy

For ICC, CHO and LoVo cell lines were reseeded into 8-well chamber slides (BD Biosciences) and allowed to adhere overnight. Cells were then washed, fixed with 4% paraformaldehyde for 15 min, and permeabilized with 0.1% saponin for 10 min. Cells were incubated with the indicated antibodies for 1 h, rhodamine-phalloidin for 20 min, and TO-PRO-3 nuclear counterstain for 5 min. For ICC of intestinal crypt organoids, organoids were collected, fixed for 30 min, and washed from residual Matrigel. Permeabilization and staining were performed in 0.2-ml tubes. Organoids were mounted onto slides using Vectashield (Vector Laboratories). Confocal microscopy images were collected and analyzed using the Leica TSC SP5 system and LAS AF Lite software. All experiments were performed at least three times.

### Wound and cell–cell adhesion assays

For the wound healing assays, CHO or LoVo cells were plated in 12-well plates, grown to near confluence, and starved in serum-free medium overnight. The next day cells were scratched, and serum-free medium was replaced with 10% serum. Wound closure was observed over a 12–20-h period, and images were captured and quantified using ImageJ (58). Cell–cell adhesion assays were performed by resuspending CHO or LoVo cells in serum-free medium and incubated with 5 μm calcein-AM dye for 30 min at 37 °C. After incubation, non-incorporated calcein-AM was removed by three washes with serum-free medium. 1 × 10^4^ calcein-AM-labeled cells were added to a confluent monolayer of CHO or LoVo cells grown in a 96-well plate. After 1- (LoVo) or 2-h (CHO) incubation at 37 °C, non-adherent calcein-AM-labeled cells were removed by washing with PBS. Relative fluorescence intensity of the adherent calcein-AM-labeled cells was measured using a Tecan M1000 plate reader with an excitation wavelength of 485 nm and an emission wavelength of 530 nm. All experiments were performed at least three times with triplicates or quadruplicates in each experiment. Data were analyzed using GraphPad Prism software.

### Quantification and statistical analyses

All quantifications of Western blots and confocal images were performed using ImageJ (58) to measure integrated density or relative length (CHO cells). Cortical actin was quantified by averaging the integrated density/pixel across a five-pixel length (*n* = ∼20 cells or crypts). For LoVo cells, IQGAP1 and β-catenin were quantified by measuring the integrated density of membrane and cytoplasm, respectively (*n* = 20–30 cells for each cell line). For each organoid type, the number of cells with wild-type junctional β-catenin expression was counted for the first 20 cells of each crypt starting at the base, averaged, and presented as a percentage (*n* = 15–20 crypts). Student's *t* test and analysis of variance were performed using GraphPad Prism.

## Author contributions

K. S. C., X. G., J. Y., L. W., A. T., C. M. M., I. M., D. J. T., K. A. M., and Q. J. L. acquired and analyzed data. K. S. C. and Q. J. L. conceived and designed the study and wrote the manuscript.

## Supplementary Material

Supplemental Data

## Footnotes

3 The abbreviations used are:

LGR5: leucine-rich repeat-containing G protein–coupled receptor 5
RSPO: R-spondin
IQGAP1: IQ motif-containing GTPase-activating protein 1
ECD: extracellular domain
7TM: seven-transmembrane
GPCR: G protein–coupled receptor
APC: adenomatous polyposis coli
ICC: immunocytochemistry
AA: amino acids
BRET: bioluminescence resonance energy transfer
GEF: guanine nucleotide exchange factor
IP: immunoprecipitation
GRD: RasGAP-related domain
PBD: p21-binding domain
PMA: phorbol 12-myristate 13-acetate
WB: Western blotting
KD: knockdown
pen/strep: penicillin/streptomycin
GAP: GTPase-activating protein.
