# Supplementary material for: LGR5 receptor promotes cell–cell adhesion in stem cells and colon cancer cells via the IQGAP1–Rac1 pathway
Source: J Biol Chem. 2017 Jul 24;292(36):14989–5001. doi: 10.1074/jbc.M117.786798 (PMC5592675; doi:10.1074/jbc.M117.786798)
Supplement: Supplemental Data [file supp_292_36_14989__index.html]

LGR5 receptor promotes cell-cell adhesion in stem cells and colon cancer cells via the IQGAP1 -Rac1 pathway — LGR5 receptor promotes cell–cell adhesion in stem cells and colon cancer cells via the IQGAP1–Rac1 pathway — LGR5 regulation of cell–cell adhesion — Supplemental Data 

# LGR5 receptor promotes cell–cell adhesion in stem cells and colon cancer cells via the IQGAP1–Rac1 pathway

## Supplemental Data

- supplementary figures (.pdf, 278 KB) - Supplementary Figures S1-3
