# Supplementary material for: LGR5 receptor promotes cell–cell adhesion in stem cells and colon cancer cells via the IQGAP1–Rac1 pathway
Source: J Biol Chem. 2017 Jul 24;292(36):14989–5001. doi: 10.1074/jbc.M117.786798 (PMC5592675; doi:10.1074/jbc.M117.786798)
Supplement: Supplemental Data [file 10.1074_M117.786798_jbc.M117.786798-1.pdf]

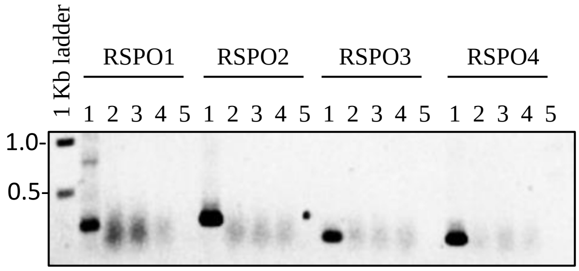

Supplementary Figure S1. RSPO expression in CHO cells and the effect of LGR5-IQGAP1 interaction in HEK293 cells. (A) Results of RT-PCR analysis of RSPO1-4 in CHO cell lines. The lanes are: 1, plasmid DNA containing the corresponding RSPO gene (positive control); 2, CHO cDNA; 3, CHO-LGR4 cDNA; 4, CHO-LGR5 cDNA; 5, no template..

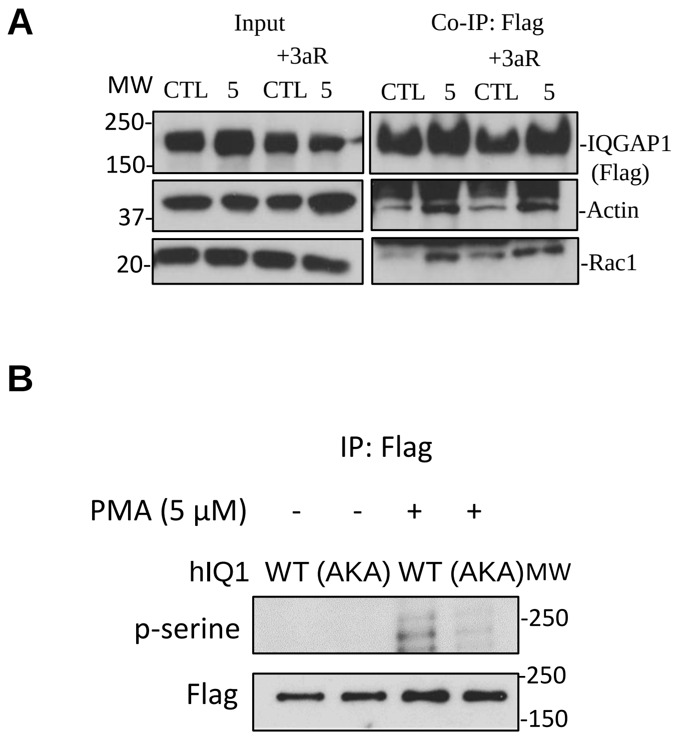

Supplementary Figure S2. The Effect of LGR5-IQGAP1 interaction in HEK293T cells. (A)). WB results of LGR5 of Co-IP analyiss of IQGAP1 with actin an Rac1 in response to Wnt3a/RSPO1 treatment. (B) Immunoprecipitation of flag-tagged wildtype (WT) or SKS→AKA Ser-1441/1443 mutant (AKA) human IQGAP1 from HEK293T cells treated +/- 5 µM PMA for 15 min at 37°C.

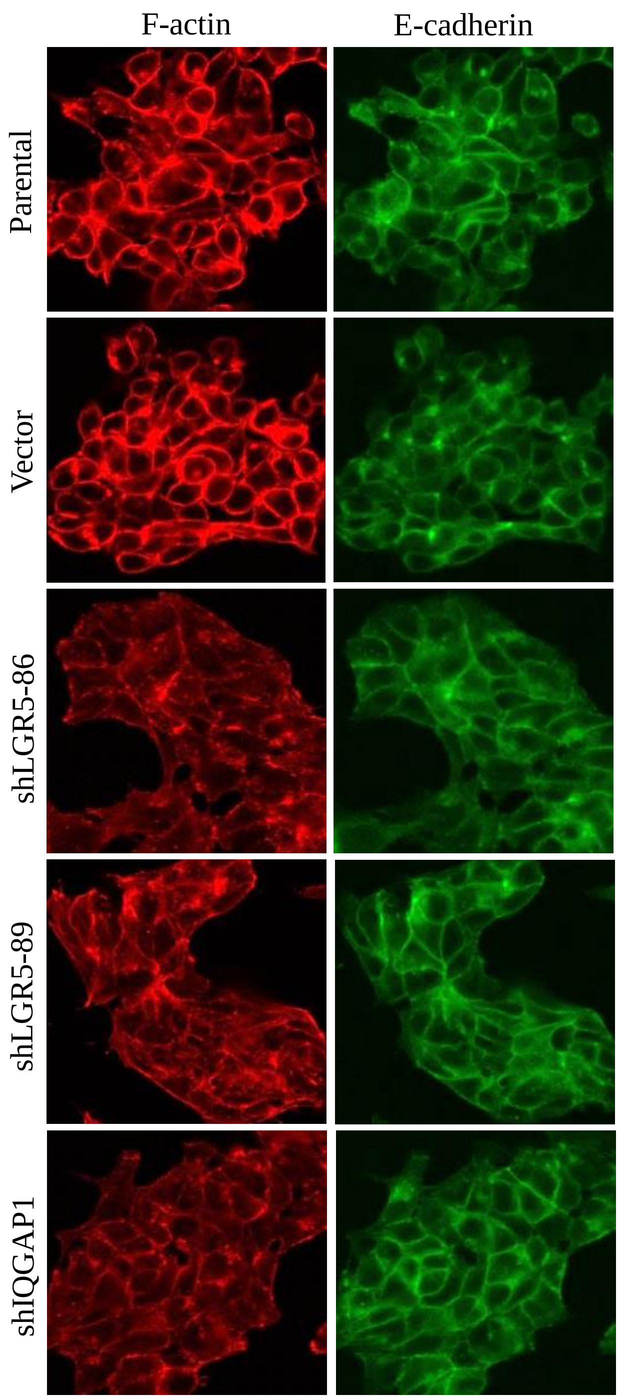

Supplementary Figure S3. Confocal microscopy images of E-cadherin (green) expression with co-staining of F-actin (phalloidin) in LoVo cells: parental, vector, and with KD of LGR5 or IQGAP1.
